# Development of a novel *PIG-A* gene mutation assay based on a GPI-anchored fluorescent protein sensor

**DOI:** 10.1186/s41021-019-0135-6

**Published:** 2019-12-10

**Authors:** Xu Tian, Youjun Chen, Jun Nakamura

**Affiliations:** 1 Department of Environmental Sciences and Engineering, University of North Carolina at Chapel Hill, Chapel Hill, NC USA; 2 Department of Neurology, UNC Neuroscience center, School of Medicine, University of North Carolina at Chapel Hill, Chapel Hill, North Carolina USA; 3 Laboratory of Laboratory Animal Science, Graduate School of Life and Environmental Biosciences, Osaka Prefecture University, Izumisano, Osaka, Japan

**Keywords:** *PIG-A*, GPI-sensor, Fluorescent protein, Genotoxicity, Gene mutation assay, Flow cytometry

## Abstract

**Background:**

Accumulation of somatic mutations caused by both endogenous and exogenous exposures is a high risk for human health, in particular, cancer. Efficient detection of somatic mutations is crucial for risk assessment of different types of exposures. Due to its requirement in the process of attaching glycosylphatidylinositol- (GPI-) anchored proteins to the cell surface, the *PIG-A* gene located on the X-chromosome is used in both in vivo and in vitro mutation assays. Loss-of-function mutations in *PIG-A* lead to the elimination of GPI-anchored proteins such that they can no longer be detected on the cell surface by antibodies. Historically, mutation assays based on the *PIG-A* gene rely on the staining of these cell-surface proteins by antibodies; however, as with any antibody-based assay, there are major limitations, especially in terms of variability and lack of specific antibodies.

**Results:**

In the current study, we developed a modified *PIG-A* mutation assay that uses the expression of GPI-anchored fluorescent proteins (henceforth referred to as a GPI-sensor), whereby the presence of fluorescence on the cell membrane is dependent on the expression of *wild-type PIG-A*. Using our modified *PIG-A* mutation assay, we have achieved complete separation of *wild type* cells and spontaneously mutated cells, in which the presence of *PIG-A* mutations has been confirmed via proaerolysin resistance and gene sequencing.

**Conclusion:**

This study establishes a novel *PIG-A* mutation assay using GPI-anchored fluorescent protein expression that eliminates the need for antibody-based staining. This GPI-sensor *PIG-A* mutation assay should be widely applicable for accurate and efficient testing of genotoxicity for use in many mammalian and vertebrate cells.

## Introduction

The accumulation of somatic mutations due to both endogenous and exogenous chemical exposures has been long known to be dangerous for human health and to cause cancer [1]. Efficient detection of somatic mutations is crucial for risk assessment of different types of exposures and is of great interest to many scientists, ranging from basic researchers to regulatory specialists. In particular, the flow cytometry-based *PIG-A* mutation assay is a commonly-used method used to detect mutations that develop in the *PIG-A* gene, which is necessary for the glycosylphatidylinositol (GPI) anchor biosynthesis pathway, by assaying for the presence of cell-surface GPI-anchored proteins [2].

The GPI anchor is a class of glycolipid structures that anchor 10–20% of cell-surface proteins to the plasma membrane and is synthesized in the endoplasmic reticulum in a pathway consisting of 11 cascading steps. A total of 22 GPI anchor synthesis proteins (GASPs) are involved in GPI anchor synthesis, such as PIG-A, PIG-C, PIG-K, PIG-O, and PIG-S, among others [3]. The *PIG-A* gene is the only GASP encoded by a gene located on the X-chromosome and encodes a critical GASP responsible for catalyzing the first step of GPI anchor synthesis [4]. In fact, the function of PIG-A was first characterized after the identification of a mutation in patients with paroxysmal nocturnal hemoglobinuria [5, 6]. In these patients, loss-of-function mutations in *PIG-A* result in the elimination of cell-surface GPI-anchored proteins, leading to the destruction of red blood cells by the complement system and ultimately intravascular hemolytic anemia [5–7].

More specifically, GPI-anchored proteins are first synthesized as precursors. The N-terminal hydrophobic signal peptide (N-SP) targets the newly-synthesized protein to the endoplasmic reticulum lumen where the N-SP is cleaved by a signal peptidase. The C-terminal GPI anchor signal sequence (C-SP) is also cleaved, and the resulting mature protein is attached to the GPI-anchor by an amide bond via a transamidase [8]. In the absence of GPI-anchor synthesis, the resulting unanchored proteins cannot be targeted to the cell membrane. In this situation, the destination of the protein from the endoplasmic reticulum depends on the status of the GPI anchor synthesis. If GPI anchor synthesis is blocked prior to the incorporation of the first mannose (for example, in *PIG-A* deficient mammalian cells), the hydrophobic C-SP will not be cleaved and the protein will be recognized as an unfolded protein and degraded by the proteasomal degradation pathway. If GPI anchor synthesis is blocked after the incorporation of the mannose (for example in *PIG-O* deficient chicken DT40 cells), the C-SP will be cleaved with the help of the mannose; however, instead of getting attached to a GPI anchor, the unanchored protein will be secreted [9]. As a result, deficiencies in the GPI anchor synthesis pathway can manifest in either the degradation (*PIG-A* mutation in mammalian cells) or the secretion (*PIG-O* mutation in chicken cells) of the unanchored proteins.

Taking advantage of the fact that *PIG-A* functional mutations can be easily detected by the absence of GPI-anchored proteins on the cell surface, in vivo and in vitro *PIG A* mutational assays have been developed, whereby the loss of cell surface GPI-anchored proteins is detected via antibody staining [10–16]. The in vivo *Pig-a* mutation assay has been used in many laboratories for basic science as well as regulatory science [17–26]. Now, a new Organization for Economic Cooperation and Development (OECD) test guideline in vivo *Pig-a* mutation assay are under preparations [17]. However, as with any antibody-based assay, this method is contingent upon the availability of sensitive and specific antibodies. In the current study, we have bypassed the limitations of antibody-based assays by constructing a GPI-sensor in which the fluorescent proteins GFP and mCherry were fused to the signal peptides at the N- and C-termini. The presence of GFP and mCherry on the cell surface is dependent on a functional GPI anchor synthesis pathway, and loss-of-function mutations in *PIG-A* lead to the absence of fluorescent signal. This modified, GPI-sensor *PIG-A* mutation assay should be widely relevant for many applications, in both mammalian cells (for *PIG-A* mutations) and in the widely-used chicken DT40 cell lines (for *PIG-O* mutations).

## Materials and methods

### Chemicals and reagents

The following chemicals and reagents were used in the study: Methyl methanesulfonate [66–27-3] (MMS) (Sigma), polybrene [28728–55-4], puromycin [53–79-2] (Sigma), TRIzol® RNA Isolation Reagent (Invitrogen), RPMI 1640 culture medium (Invitrogen), penicillin/streptomycin (Invitrogen), fetal bovine serum (Atlanta Biologicals), iScript™ cDNA Synthesis Kit (Bio-Rad Laboratories, Inc.), Taq DNA Polymerase (New England Biolabs.), TransIT®-293 transfection reagent (Mirus Bio LLC) and Fixable Viability Stain 450(FVS450) (BD Biosciences).

### Lentiviral-mediated cDNA expression

The *GFP* and *mCherry* genes were inserted into the pUltra vector, along with the translocation signal sequence of acrosin on the 5′ end and the CD90 GPI anchor signal sequence in the 3′ end, all under the control of the hUBC promoter [27]. Lentiviral particles were prepared and target cells were infected according to a standard protocol, as previously used in our lab [28]. Briefly, 2 μg of pUltra GPI-GFP or GPI-mCherry, 2 μg of pREV, 2 μg of pGag/Pol and 1 μg of pVSVG were transfected into HEK293T cells in a 6 cm dish using the TransIT®-293 transfection reagent. Cell culture medium was changed 24 h later, and the supernatant containing lentiviral particles was collected 2 days after transfection. TK6 cells were infected with lentivirus in the presence of 5 μg/ml polybrene, and after 24 h, fresh medium was replaced. Two to three days post-transduction, the expression of the fluorescent proteins was detected via fluoresce microscope and flow cytometry.

### Cell culture

Human B-lymphoblastoid TK6 cells were maintained in our lab and were cultured in RPMI-1640 medium supplemented with 10% fetal bovine serum and 1% penicillin/streptomycin. HEK293T cells were purchased from the Lineberger Comprehensive Cancer Center at the University of North Carolina at Chapel Hill and were cultured in DMEM medium supplemented with L-glutamine, 10% fetal bovine serum and 1% penicillin/streptomycin. All cells were maintained at 37 °C with 5% CO2.

### Flow cytometry for cell sorting and detection

GFP and mCherry double positive TK6 cells were sorted using a FACSAria II (BSL2) (UNC Flow Cytometry Core Facility) using a standard protocol. Briefly, cells were harvested and washed with PBS and then placed in sorting buffer (HEPES-buffered HBSS with 1% BSA) at 10^7^ cells/ml. Target cells were sorted into collection buffer (HEPES-buffered RPMI 1640 medium with 10% fetal bovine serum).

Flow cytometry analyses were performed with an LSRFortessa (BSL2) (UNC Flow Cytometry Core Facility). Cells were collected and washed with PBS. After staining with the BD Horizon™ Fixable Viability Stain 450 (FVS450) for 15 min at room temperature using a standard protocol, the cells were washed with PBS and filtered before flow cytometry analyses. Excitation and emission detection wavelengths of the fluorescence were the following: FITC (488–530/30 nm), mCherry (561–610/20 nm), FVS450 (405–450/50 nm). Samples were analyzed at ~ 8000 events/second. More than 10^6^ cells were collected in order to detect GPI(−) frequency.

### Spiking experiment

Spiked cell samples were prepared by mixing 0, 10, 20, 40, 80 GPI(−) cells into every 10^6^ freshly re-populated GPI(+) cells.

### Determination of the phenotypic expression time for TK6 cells

To determine the phenotypic expression time for TK6 cells, GPI(+) cells were treated with 12 and 24 μM MMS for 24 h, and GPI(−) cells were detected via flow cytometry every 2 days from day 8 to day 14.

### MMS treatment

3 × 10^6^ cells were incubated in 10 ml medium with MMS. After 24 h of exposure, cells were washed with PBS and 2 × 10^6^ cells were subcultured every other day into 25 ml of medium.

### *PIG-A* sequencing

cDNA was prepared as previously described [28]. Briefly, total RNA was isolated by using TRIzol® RNA Isolation Reagent following the manufacture’s protocol. The same amount of total RNA was used for reverse transcription using the iScript™ cDNA Synthesis Kit following the manufacture’s protocol. Taq DNA polymerase and the following primers were used to amplify *PIG-A*: F1: 5′ > ggttgctctaagaactgatgtc < 3′; R1: 5′ > atcatgccttctaaatgggtc< 3′; F2: 5′ > agcttctaaccgtgtctctt < 3′; R2: 5′ > cccccaaaagcaaggttatt< 3′. For F1&R1, 64 °C was used for the annealing temperature, and for F2&R2, 56 °C was used. The primer set F1 and R3: 5′ > tcttacaatctaggcttccttc< 3′ was used for sequencing. The *PIG-A* gene sequence from freshly re-populated GPI(+) cells was used as a *wild-type* reference.

## Results

### Design and expression of fluorescent sensors on the cell surface

**Fig. 1:**
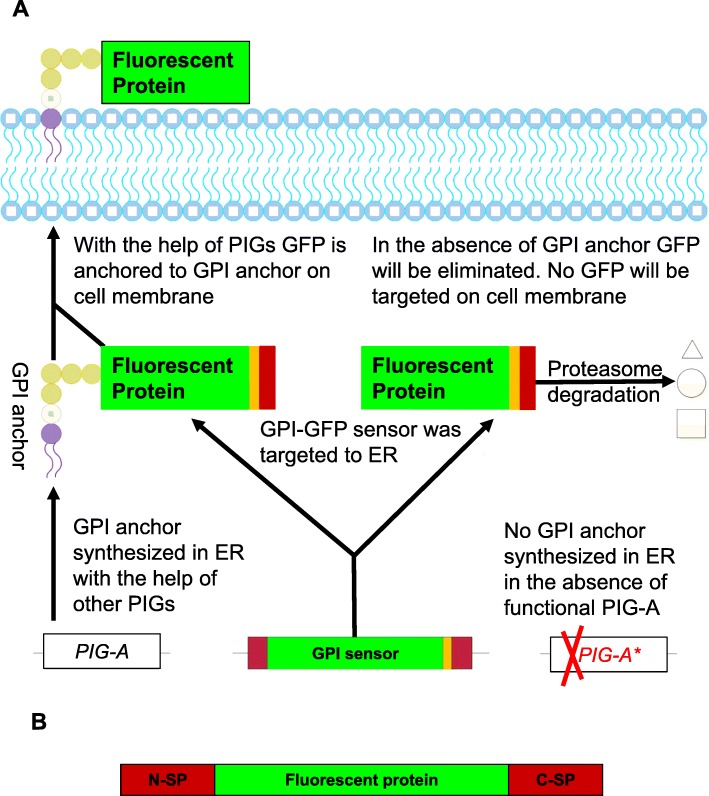
Fluorescent GPI-sensor *PIG-A* mutation assay schematic and GPI-anchored protein precursor structure. **a** GPI-sensor *PIG-A* mutation assay schematic. **b** GPI-anchored protein precursor structure

The conventional *PIG-A* mutation assay involves measuring levels of endogenous GPI-anchored proteins on the cell surface using antibodies specific for these proteins [12–16]. In this study, we wanted to determine whether we could artificially express and tether proteins on the cell surface via a GPI anchor. To address this question, we designed constructs for GPI-anchored fluorescent proteins (Fig. 1). The N-terminus of the fluorescent protein was tagged with a hydrophobic signal peptide (N-SP) that targeted the fluorescent protein to the endoplasmic reticulum lumen. The C-terminal signal peptide (C-SP) was also added for the addition of the GPI-anchor via post translational modification [8]. Successful detection of the resulting mature fluorescent protein at the cell surface indicates a functional, intact GPI-anchor synthesis pathway. On the other hand, if there is any malfunction in the GPI-anchor synthesis pathway, fluorescent proteins would either be degraded or secreted outside of cells such that no fluorescence at the cell surface would be observed.

Theoretically, when using a GPI-sensor to detect the presence of functional mutations in the *PIG-A* gene, a mutation in the sensor itself could interfere with the assay. To address this potential issue, we expressed two fluorescent sensors in each cell. If the fluorescent sensor mutation rate is similar to the *PIG-A* spontaneous mutation rate, which is around 10^− 6^, the ratio of cells that develop mutations in both fluorescent sensors will be around 10^− 12^, which is a rate that can deemed as negligible in the *PIG-A* mutation assay. In our assay, we co-expressed GPI-GFP and GPI-mCherry in our cells, and only double negative cells were deemed to have a *PIG-A* mutation.

**Fig. 2:**
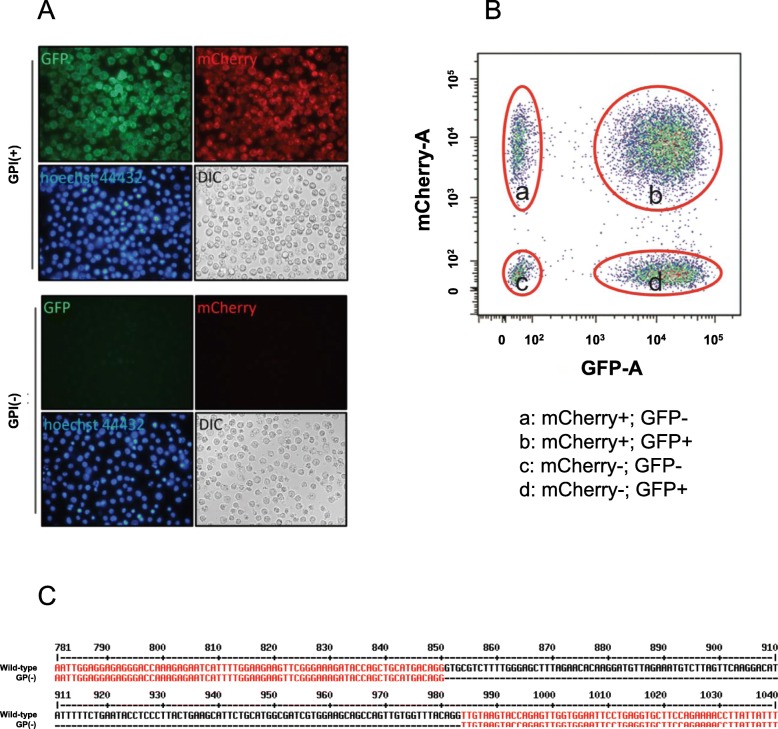
Expression of GPI-anchored GFP/mCherry. **a** Lentiviruses expressing GPI-anchored GFP and mCherry (fluorescent GPI-sensors) were prepared, and TK6 cells were infected. The expression of the GPI-sensors in TK6 cells were confirmed using an inverted fluorescent microscope. GPI-GFP and GPI-mCherry were confirmed to be targeted to the cell membrane. Proaerolysin (PA) resistant cells were labeled as GPI(−) cells, and the expression of GPI-sensors were compared under the fluorescent microscipe with GPI(+) cells. **b** The expression of GPI-sensors in TK6 cells were also confirmed by using flow cytometry using an LSRFortessa (BSL2). Signal intensity was measured by area on the histograms. Four group of cells can be detected: GFP and mCherry double-negative cells, GFP- or mCherry-single positive cells, and double positive cells. GFP and mCherry double positive TK6 cells were sorted by FACSAria II (BSL2), and single cells were expanded as GPI(+) cells for following experiments. **c** GPI(−) clonal cells were isolated, and the *PIG-A* gene was sequenced and compared with *wild-type*

We constructed GPI-anchored GFP and GPI-anchored mCherry lentiviral expression vectors and infected TK6 cells with the GPI-anchored GFP and GPI-anchored mCherry lentiviruses using a standard protocol [28]. To confirm the expression of GFP and mCherry, we examined the cells infected with the lentiviruses under a fluorescent microscope and found that both GFP and mCherry were present on the cell surface (Fig. 2a). Next, we determined if we could separate GFP and mCherry double-positive cells from single-positive and double-negative cells by flow cytometry using an LSRFortessa (BSL2). The four cell populations were clearly separated (Fig. 2b, Groups (a)-(d)). We further sorted GFP and mCherry double-positive cells (Fig. 2b, Group (b)) by FACSAria II (BSL2) and cultured them for further experiments as GPI(+) cells.

### Fluorescent signals are absent in cells carrying *PIG-A* mutation

To determine if the expression of GPI-GFP or mCherry on the cell surface is indeed dependent on intact PIG-A function, we first generated cells carrying *PIG-A* mutations. We treated TK6 cells with proaerolysin (PA), which kills off cells with GPI-anchored proteins on the cell surface (i.e. cells without *PIG-A* mutations). The remaining PA-resistant cells are those that have spontaneous *PIG-A* mutations [3]. We infected *wild-type* and *PIG-A* mutant cells with the GPI-GFP and mCherry lentiviruses and examined the fluorescent signal. We found that, unlike *wild-type* cells, no fluorescent signal could be detected in *PIG-A* mutant cells (Data not shown). To eliminate the possibility that a *PIG-A* deficiency could suppress viral infection, we treated GPI(+) cells that initially expressed both GFP and mCherry on the cell surface with PA and selected the resistant cells that developed spontaneous *PIG-A* mutations. We found that GFP and mCherry were no longer expressed on the surface of these cells (Fig. 2a). These results suggest that in the absence of GPI anchor synthesis (i.e. in cells with a *PIG-A* mutation), GPI-GFP/mCherry is degraded or secreted, and there is no fluorescent signal maintained in the cells. We sorted these cells and maintained them as GPI(−) cells for further experiments.

To confirm the presence of a mutation in the *PIG-A* gene in GFP and mCherry double negative cells, we sorted the cells by FACSAria II and cultured single clone cells. Ten clonal populations of cells were exposed to PA and were found to be resistant, indicating the presence of mutation in *PIG-A*. Additionally, sequencing the *PIG-A* gene of those clonal cells identified the same deletion mutation in all ten clones (Fig. 2c).

### Reduction of a spontaneous *PIG-A* mutation by repopulation

**Fig. 3:**
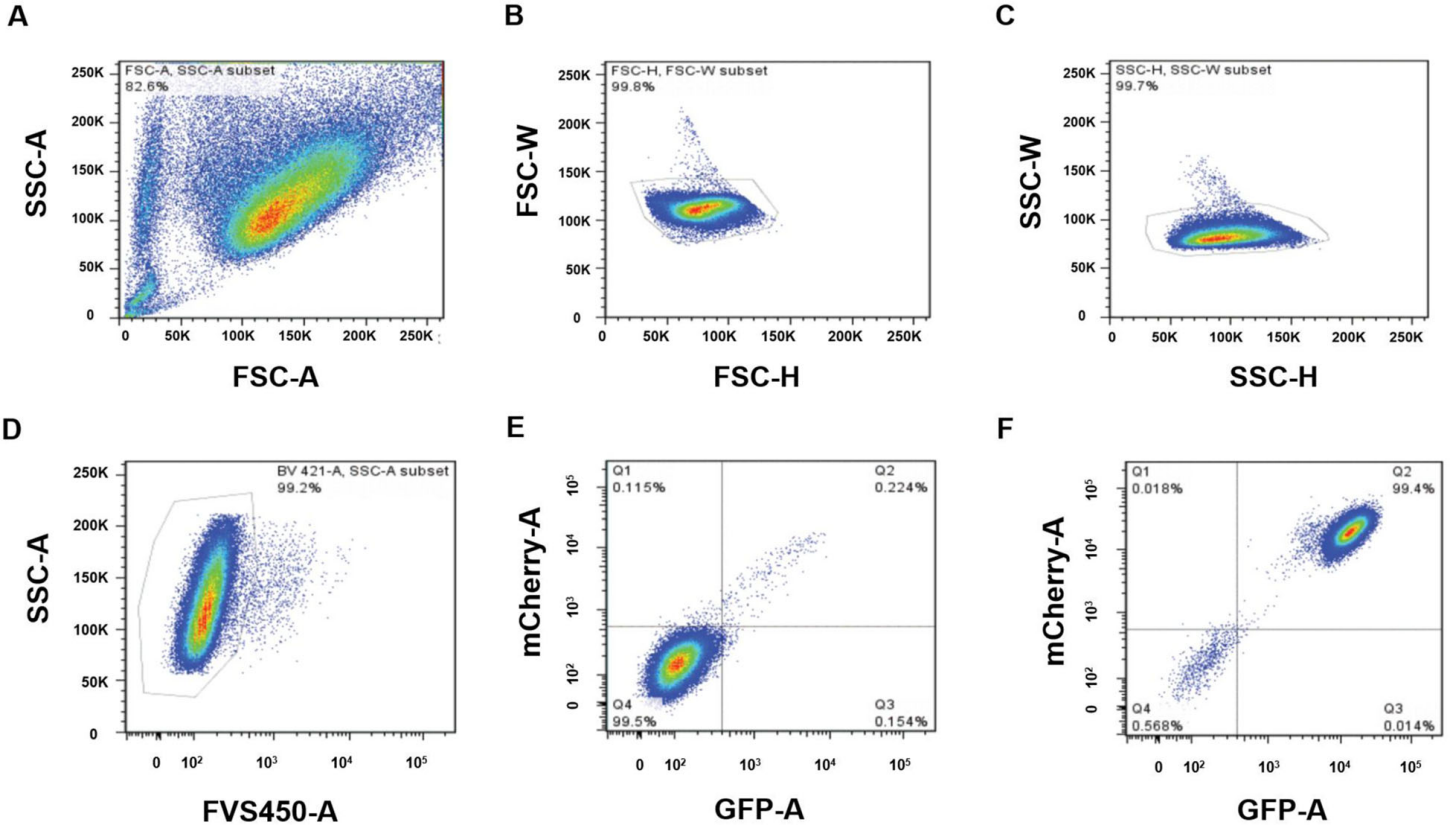
Flow cytometric analysis of GPI(−) cell frequency. Flow cytometry was performed using an LSRFortessa (BSL2). **a** Cells were collected by FSC/SSC, **b, c** Single cells were collected via FSC-W/FSC-H and SSC-W/SSC-H. **d** Dead cells were eliminated by FVS450 staining. GPI(−) (**e**) and GPI(+) (**f**) cell numbers were measured, and spontaneously mutated cells were isolated in (**f**) Q4

The gating procedure for flow cytometry analysis using the LSRFortessa is shown in Fig. 3. Cells were collected by FSC/SSC gate scatter (Fig. 3a), and single cells were collected by FSC-W/FSC-H and SSC-W/SSC-H (Fig. 3b and c). Dead cells were excluded via FVS450 staining (Fig. 3d). GPI(−) cells were used as a negative control (Fig. 3e). GPI(+) cells were measured, and *wild-type* cells (Fig. 3f, Q2) and spontaneous mutation cells (Fig. 3f Q4) were separated.

**Fig. 4:**
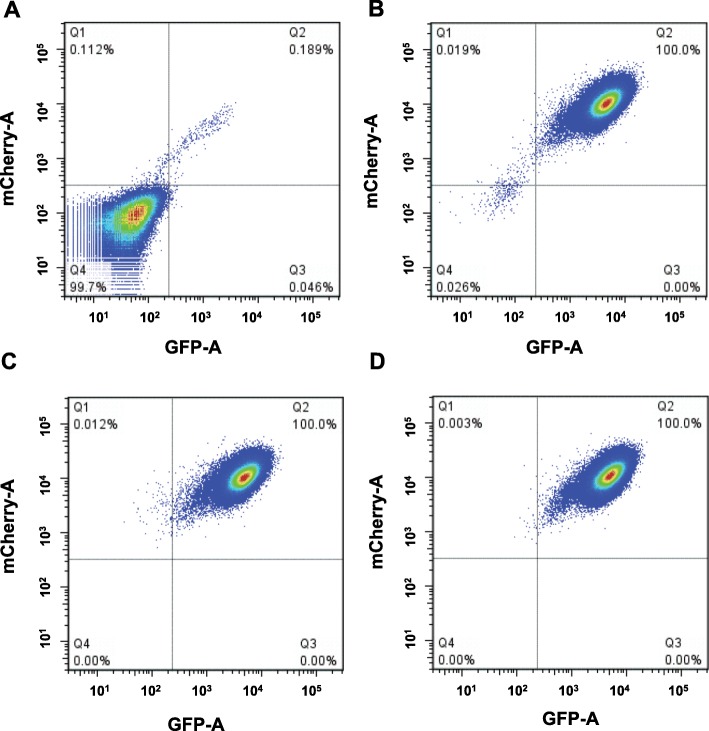
Depletion of pre-existing GPI(−) TK6 cells by repopulation. Flow cytometry was performed using an LSRFortessa (BSL2). GFP/mCherry double positive GPI(+) cells located in Q2, and GFP/mCherry double negative GPI(−) cells located in Q4. **a** GPI(−) cells and **b** GPI(+) cells before repopulation of GPI(+) cells using fluorescence-activated cell sorter, **c, d** two GPI(+) cells after repopulation of GPI(+) cells using fluorescence-activated cell sorter

There was a small amount of cells carrying spontaneous *PIG-A* mutations which can accumulate over time or in the process of cell expansion (Fig. 4b). The presence of these GPI(−) cells can reduce the consistency and sensitivity of this mutation assay, so it is necessary to exclude these cells. To accomplish this, we repopulated 4000–5000 cells (based on an actual negative cell rate) in multiple dishes. Afterward, we obtained some dishes with low preexisting GPI(−) cells. Two examples of repopulated cells were shown Fig. 4c and d.

### Detection of rare GPI(−) cells

**Fig. 5:**
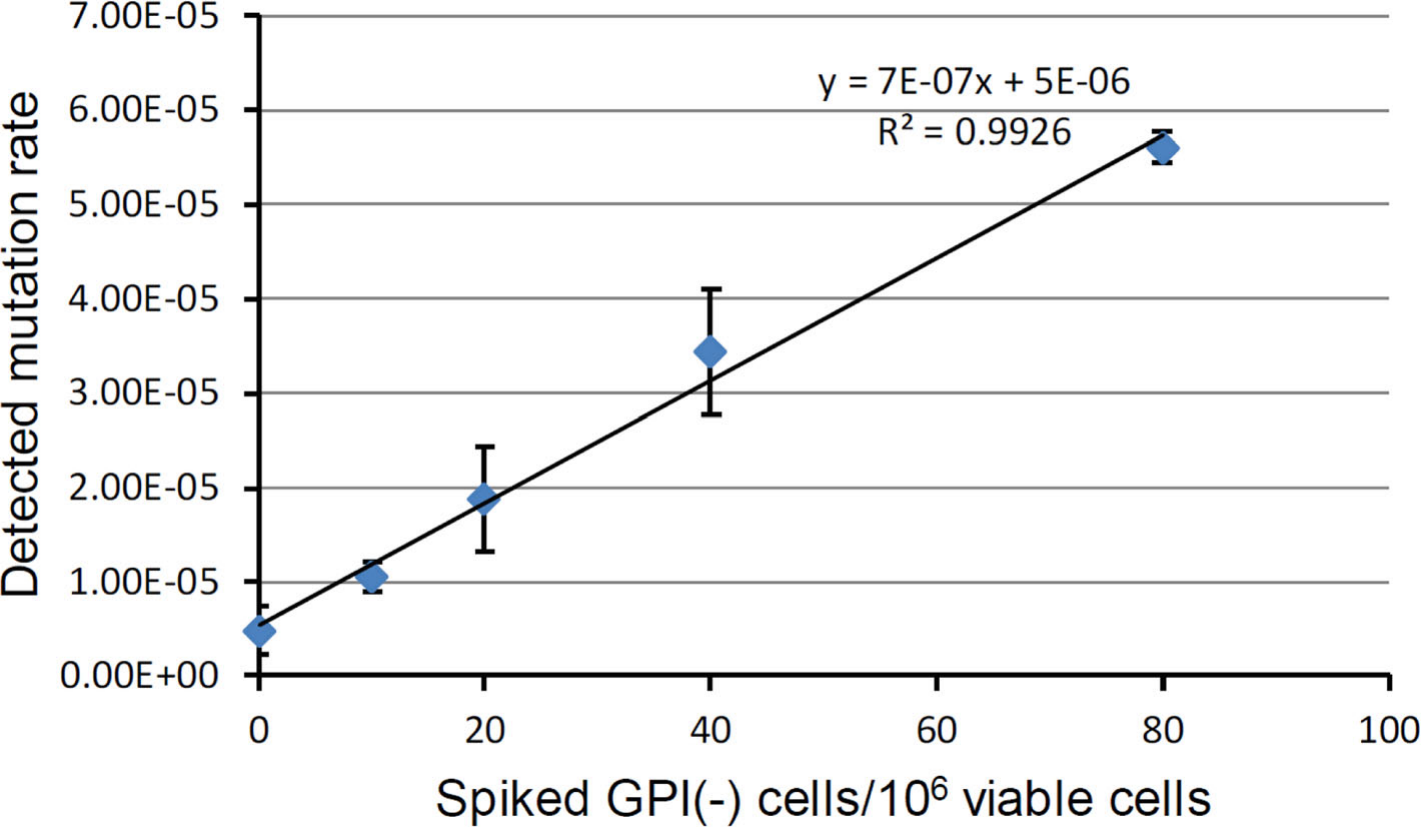
Recovery of spiked GPI(−) TK6 cells via flow cytometry. 0, 10, 20, 40 and 80 GPI(−) cells were spiked into 1 × 10^6^ repopulated GPI(+) cells, and the mutation rate (GPI(−) cells) were detected via flow cytometry. Shown are mean value ± SD for three independent samples for each spiking level

It is necessary that we are able to detect rare mutations from a large pool of wild type cells. To determine the sensitivity of this assay, we performed spiking experiment, in which we artificially mixed a few GPI(−) cells with a large amount of GPI(+) cells and examined if similar amount of GPI(−) cells could be detected using flow cytometry. We found that the ratio of spiked GPI(−) cells correlated linearly with the mutation rate (Fig. 5), suggesting that this GPI-sensor *PIG-A* mutation assay is sufficient to recover and quantify vary rare GPI(−) cells.

### Optimization of GPI(−) phenotypic expression period

**Fig. 6:**
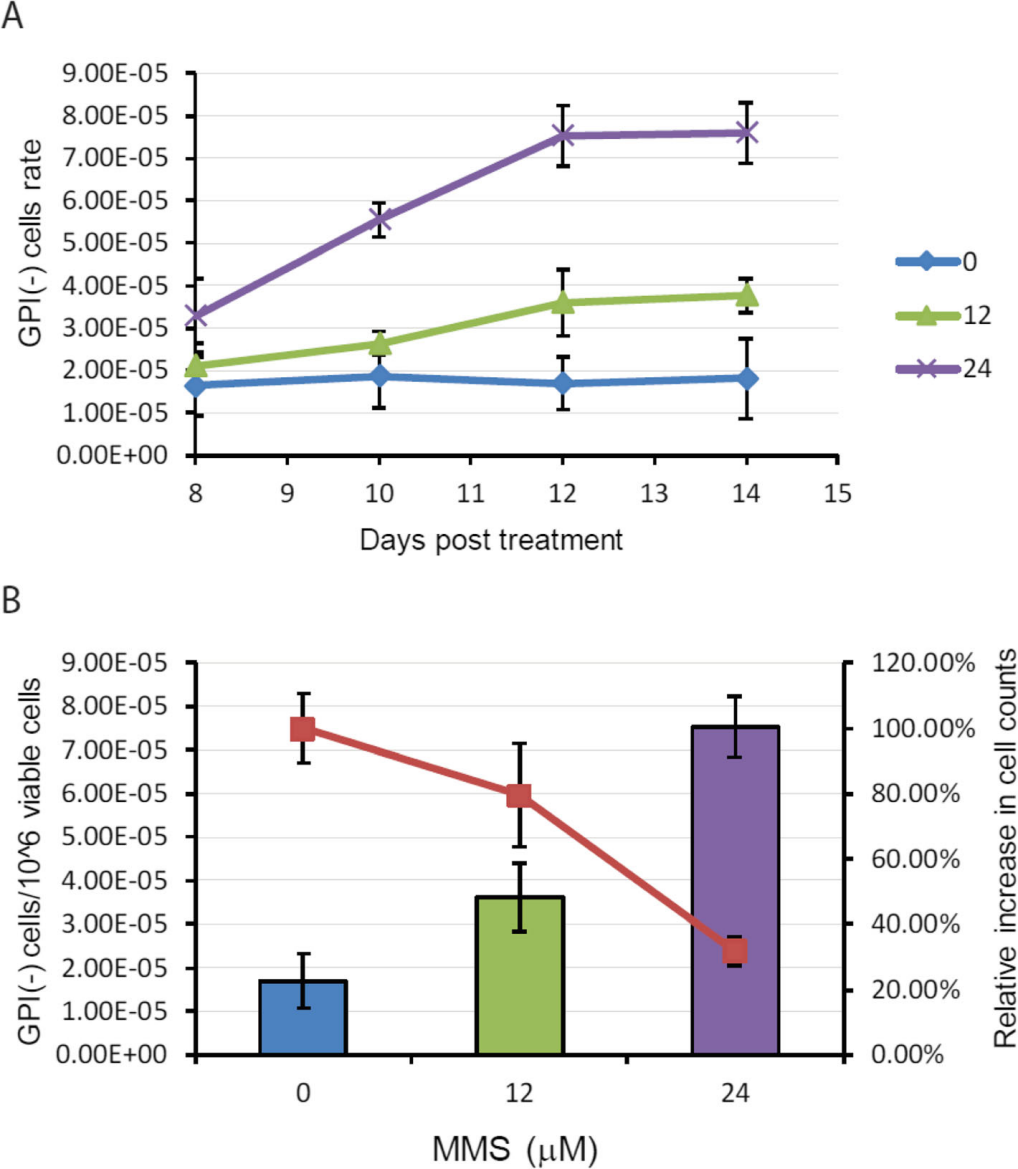
Detection of mutations after MMS treatment. **a** GPI(−) phenotypic expression time for TK6 cells. Cells were treated with 0, 12 or 24 μM MMS for 24 h, washed and cultured in fresh medium. GPI(−) frequency was detected by flow cytometry on days 8, 10, 12 and 14 after exposure. Shown are mean value ± SD for three independent samples. GPI(−) cells frequency after 24 μM MMS exposure (**b**). Bars represent the GPI(−) frequency and lines represent relative increases in cells counts

Next, we determined the optimal GPI(−) phenotypic expression period for TK6 cells for the GPI-sensor *PIG-A* mutation assay. The phenotypic expression period is the time required for mutated cells to have the *wild-type PIG-A* mRNA, protein and existing GPI-anchored proteins to be either degraded or diluted to allow for the manifestation of the mutation phenotype. To determine the optimal GPI(−) phenotypic expression period, we exposed TK6 cells with water, 12 μM or 24 μM of MMS for 24 h before collecting samples. Frequency of GPI(−) cells was measured every two days by flow cytometry from day 8 to day 14 (Fig. 6). As expected, cells treated with water did not show any significant change in the frequency of GPI(−) cells (Fig. 6a, blue line). Exposure of cells to either 12 μM or 24 μM of MMS showed an initial increase in the ratio of GPI(−) cells, which peaked at around day 12, after which the mutational frequency plateaued. Thus, the phenotypic expression period for TK6 cells for GPI-sensor *PIG-A* mutation assay was set at 12 days.

### Fluorescent sensor assay effectively detects mutation in cells post-MMS exposure

Finally, we measured the ratio of cells harboring *PIG-A* mutations and cell proliferation as a result of MMS exposure. To do this, GPI(+) TK6 cells were treated with water, 12 μM or 24 μM MMS. Cell numbers were counted every two days, and relative increases in cell counts were calculated accordingly. The *PIG-A* mutation rate was measured on day 12 after MMS exposure. We found that cells treated with 24 μM MMS contained the highest ratio of GFP(−) cells compared to control or 12 μM MMS treatment (Fig. 6b). At the same time, we found that MMS treatment suppressed cell proliferation. On day 12, the total number of cells after 24 h of MMS treatment was only 25% that of control treatment.

## Discussion

In the current study, we have successfully established a novel *PIG-A* mutation assay that takes advantage of the GPI anchor synthesis pathway. We generated GPI-GFP and GPI-mCherry double expressing TK6 *wild-type* cells and confirmed their membrane localization via fluorescent microscopy. The absence of GFP and mCherry fluorescence in *PIG-A* mutation cells indicated the lack of cell surface GPI-anchored protein expression, which was confirmed by PA resistance and *PIG-A* sequencing. A spiking experiment shows that our fluorescent GPI-sensor is sufficient to detect very low frequency of GPI(−) negative cells. Finally, we exposed TK6 cells to MMS, determined the phenotypic expression time, and examined genotoxicity induced by MMS using our newly established fluorescent GPI-sensor *PIG-A* mutation assay.

Conventional *PIG-A* mutation assays are contingent upon antibodies that are sufficiently sensitive and specific for the detection of endogenous GPI-anchored proteins. This antibody-based assay can be expensive and time-consuming. In some conditions, suitable antibodies may not be available for certain cell lines. To avoid the caveats of antibody staining which may result non-specific labelling or inconsistent measurements, we engineered artificial GPI-anchored fluorescent proteins, GPI-GFP and GPI-mCherry, that are tethered to the membrane. Mutations in *PIG-A* lead to a deficiency in GPI anchor synthesis and, consequently, an absence of fluorescent signal. Our fluorescent GPI-sensor *PIG-A* mutation assay completely eradicates the need for antibody staining, which not only saves time and resources but also extends the use of the *PIG-A* mutation assay to a broader field of cells types and species. In theory, any eukaryotic cell which has one copy of any critical GASP (where one functional mutation in that GASP will cause phenotype changes) will be suitable for our fluorescent GPI-sensor mutation assay.

For example, we previously attempted to establish a flow cytometry-based *PIG-O* mutation in DT40 cells [3]; however, we were unable to find suitable antibodies or a specific dye to distinguish GPI(−) cells from GPI(+) cells. Recently, we applied our fluorescent GPI-sensor assay to the DT40 *PIG-O* mutation assay. We found that our GPI-sensor is compatible with DT40 cells, and our preliminary data show successful expression of the GPI-sensor in DT40 cells (data not shown). With minor optimizations, our fluorescent GPI-sensor should also work with the DT40 *PIG-O* mutation assay.

To confirm the presence of mutations in the *PIG-A* gene, we sequenced ten clonal spontaneously mutated PIG(−) cells. To our surprise, all of the clones showed the same deletion mutation as PIG(+) cells. This may be due to the possibility that the mutation occurred at a relatively early stage when we repopulated the cells.

Of note, it has previously been reported that in TK6 cells, there is a hemizygous deletion at the 17p12-p22 (275,712 bp) locus, which covers the 5′ region of the *PIG-L* gene [29]. This means that in TK6 cells, a single functional mutation in *PIG-A* or *PIG-L* will cause a loss of GPI anchor synthesis function. In the case that a mutation in the *PIG-A* gene is not detected, we will have to sequence *PIG-L* in order to study the mutation spectrum.

## Conclusions

In this study, we developed a modified *PIG-A* mutation assay utilizing GPI-anchored fluorescent protein expression that eliminates need for antibody-based staining. This GPI-sensor *PIG-A* mutation assay is compatible with many mammalian and vertebrate cells, such as DT40 cells, and should be widely applicable for accurate and efficient testing of genotoxicity.

## Data Availability

Not applicable.

## Abbreviations

C-SP: C-terminal signal peptide
FVS450: Fixable Viability Stain 450
GASPs: GPI anchor synthesis proteins
GPI: Glycosylphatidylinositol
GPI(−): GPI anchor synthesis deficient
GPI(+): GPI anchor synthesis proficient
GPI-sensor: GPI-anchored fluorescent protein
MMS: Methyl methanesulfonate
N-SP: N-terminal signal peptide
PA: Proaerolysin
